# Ultrasound-guided radiofrequency ablation for the treatment of anaplastic thyroid cancer: a case report

**DOI:** 10.3389/fonc.2025.1469167

**Published:** 2025-05-26

**Authors:** Tian Liu, Chen Xu, Hangjun Chen, Fuhua Chen, Bei Feng, Xiaoying Tao, Yibo Zhou

**Affiliations:** Department of Ultrasound, Affiliated Jinhua Hospital, Zhejiang University School of Medicine, Jinhua, China

**Keywords:** ultrasound-guided, radiofrequency ablation, anaplastic thyroid cancer, case report, radiofrequency ablation (RFA)

## Abstract

**Background:**

Anaplastic thyroid cancer (ATC) is a rare but highly aggressive tumor of the thyroid. Due to its rapid progression and resistance to radiotherapy, the prognosis of ATC is poor. Novel treatments have been increasingly applied in recent years, but the outcome of treatment is not satisfactory.

**Case presentation:**

We present a case of advanced ATC that cannot be surgically resected, who underwent ultrasound-guided radiofrequency ablation (RFA) combined with immunotherapy and targeted therapy for tumor shrinkage. The patient’s symptoms, including neck pain, were significantly relieved, and the follow-up of this patient showed a satisfactory outcome with no associated complications. To the best of our knowledge, this is the first case of palliative treatment by RFA in a patient with advanced ATC. It exemplifies a new application and success of this classical approach in palliative treatment.

**Conclusion:**

This case indicates that RFA has a good therapeutic effect on elderly patients with advanced ATC. Such an approach will provide new ideas for the treatment of advanced ATC and warrants further study in the future.

## Introduction

Anaplastic thyroid carcinoma (ATC) is a highly aggressive malignant tumor of the thyroid follicular epithelium. Due to its rapid progression and resistance to radiotherapy, ATC has a very low cure rate and a median survival of less than 6 months, accounting for 14%–50% of all thyroid cancer deaths (1, 2). Most ATC patients have extensive peripheral invasion and distant metastasis when they are found, so the prognosis is poor. At present, the conventional treatment of ATC is mainly based on the integrated treatment of surgery and radiotherapy, supplemented by systemic therapy. In recent years, new treatment methods have been increasingly applied and brought hope to treating ATC (3, 4). In this case, we present a patient with advanced ATC that cannot be surgically resected who underwent ultrasound-guided radiofrequency ablation (RFA) combined with immunotherapy and targeted therapy for tumor shrinkage and relief of neck compression symptoms.

## Case presentation

**Figure 1:**
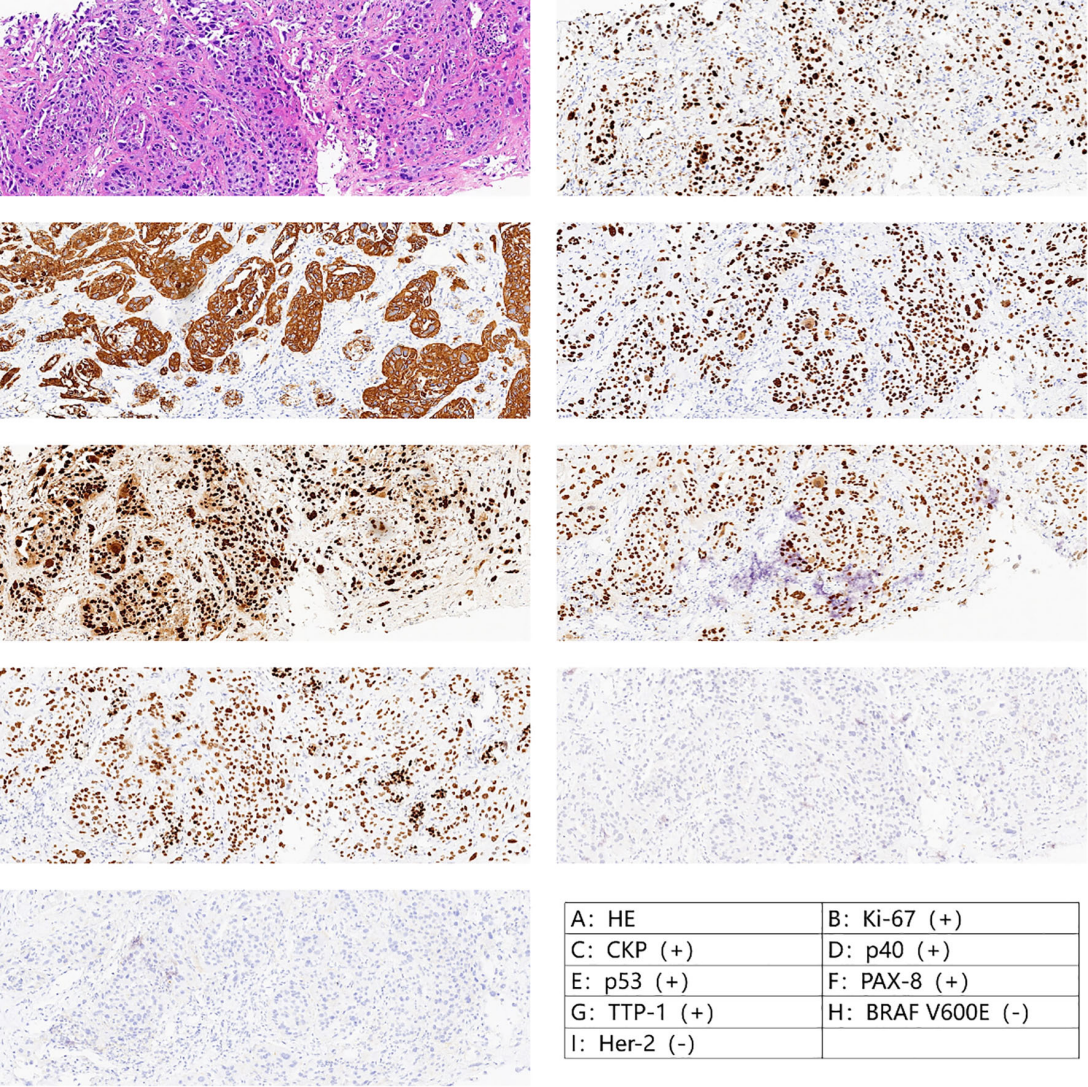
Pathology after crude needle aspiration suggested undifferentiated carcinoma with squamous differentiation.

An 84-year-old man was diagnosed with ATC. The patient came to our hospital because he found a neck mass. On examination, we found that the patient’s trachea was compressed to the left, and the right thyroid gland could be reached with a mass of about 4×5 cm in size, medium in texture, clear boundary, smooth surface, no pressure pain, and could move up and down with swallowing. The patient’s general condition was fair, with underlying diseases such as hypertension, diabetes mellitus, and coronary artery disease. Laboratory tests were unremarkable except for an elevated thyroid peroxidase antibody (56 IU/ml). The patient underwent thyroid ultrasonography, which revealed a huge thyroid mass, and then underwent a fine-needle aspiration biopsy of the thyroid (FNA). Post-operative pathology suggested low-undifferentiated carcinoma of the thyroid (TBSRTC category VI). Therefore, he underwent another thyroid coarse needle aspiration biopsy (Core Needle Biopsy, CNB), and the postoperative pathology and immunohistochemistry suggested ATC with B3-3, CK-P (+), Ki-67(+) 90%, P53(+), P40(+), Pax-8(+), TTF-1(+), BRAF V600E(I2) (–), and Her-2 (0) (**Figure 1**).

**Figure 2:**
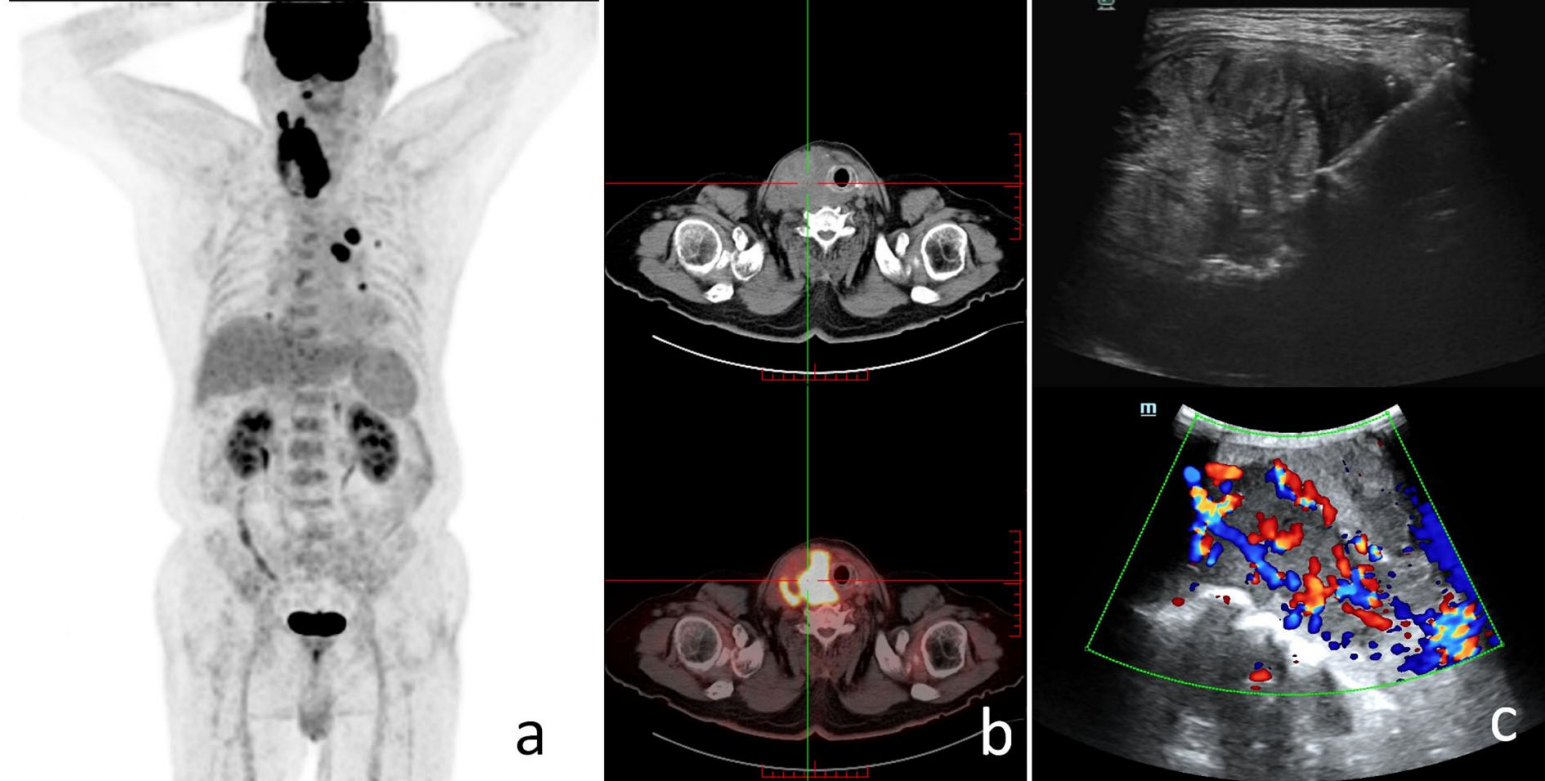
The patient's preoperative findings. **(a)** The patient's PET-CT scan showed increased FDG metabolism in the right parapharyngeal space, cervical lymph nodes, both lungs, and mediastinal lymph nodes; systemic multiple metastases were considered. **(b)** SPECT scan suggested a hypermetabolic thyroid nodule. **(c)** The patient's ultrasound imaging suggested a thyroid nodular lesion.

We also performed FNA of the abnormal lymph nodes in the neck, and the pathology suggested lymph node metastasis. The patient then underwent PET-CT whole-body tomography imaging, and the results suggested possible metastases in the right parapharyngeal space, with multiple lymph nodes in the neck, and possible multiple metastases in both lungs. In addition, metastases could not be excluded from the mediastinal group of 5 lymph nodes with increased FDG metabolism (**Figure 2**). The above findings suggested that the patient was diagnosed with right-sided low undifferentiated thyroid cancer with lymph node metastasis in the lateral neck region and possible lymph node metastasis in both lungs and mediastinum (cT4N1M1, stage 4B).

The patient was evaluated comprehensively by multidisciplinary (including thyroid surgery, ultrasound medicine, oncology, radiotherapy, nuclear medicine, etc.). A comprehensive treatment strategy was ultimately developed. Given the advanced age of the patient, the pathology of undifferentiated carcinoma with distant metastases, and the poor surgical outcome, the surgeon felt this patient should have undergone tumor reduction therapy. On the other hand, the patient and his relatives did not accept radiotherapy. After the MDT discussion, we finally suggested that the patient could try ultrasound-guided RFA combined with immunotherapy and targeted therapy (Treprostinil plus Anrotinib) of the thyroid tumor for treatment.

**Figure 3:**
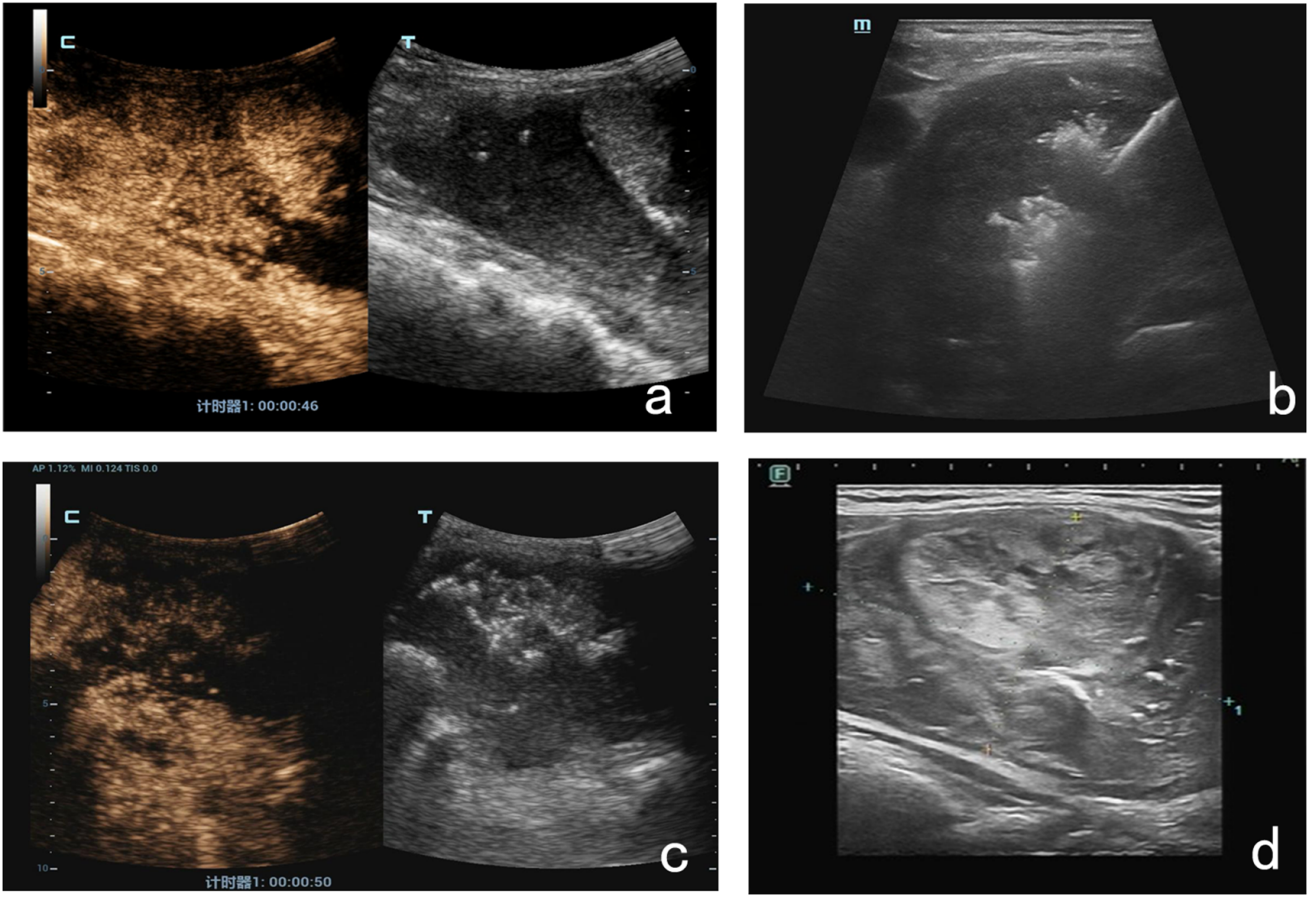
The RFA procedure and postoperative ultrasound findings. **(a)** Before RFA, the CEUS results suggested contrast filling within the lesion. **(b)** An ultrasound-guided radiofrequency needle was placed deep into the nodule. **(c)** At the end of the procedure, CEUS results suggested no significant local contrast filling of the lesion. **(d)** One month after RFA, the lesion was 20% smaller than before.

Due to the large size of the mass, we used a multiple-procedure strategy to minimize surgical risk. An ultrasound-guided radiofrequency needle was placed deep into the nodule to begin ablation. During the procedure, the unipolar/bipolar RFA electrode needle 18-07s 10F was inserted into multiple right-sided thyroid nodules, and mobile ablation was performed until the ablation area was visibly vaporized and covered approximately 50% of the mass. For protection and isolation, 10 ml of saline was injected into the gap between the right anterior thyroid envelope and the muscularis propria and with the carotid artery. A pulsed mode was used, with a power of 45w and 40w for the first procedure and the second, respectively, and mobile ablation was performed using multipoint needle entry. The total ablation time was 2 minutes and 30 seconds. At the end of the procedure, ultrasound and contrast-enhanced ultrasound (CEUS) were used to assess the efficacy of the RFA procedure. The results showed no significant contrast perfusion in the ablated area, suggesting complete ablation. We followed up on this patient in the first and third month after RFA and found that the mass had shrunk by 20–30% compared with the previous one (**Figure 3**). The patient’s symptoms, including neck pain, were also significantly relieved, and there were no other uncomfortable symptoms. The patient was willing to continue to receive maintenance treatment and further follow-up. This report followed the CARE checklist guidelines for case reports.

## Discussion

ATC accounts for 1.3–9.8% of all thyroid cancers (5). Although rare, ATC, as an aggressive tumor, grows rapidly and tends to have a poor prognosis (6). ATC often presents as a rapidly enlarging neck mass, which may lead to severe compression of the airways and clinical symptoms such as hoarseness, dysphagia, dyspnea, cough, pain, and hemoptysis. According to the staging criteria of the 8th edition of the American Joint Committee on Cancer (AJCC), all patients with ATC are stage IV upon diagnosis (7). Accordingly, curative resection at the first presentation of the tumor is often difficult. Given the rapid progression of ATC, the resectability and extent of the tumor should be determined as soon as possible by routine preoperative investigations (ultrasound, CT, MRI, or PET scanning), laryngoscopy, and bronchoscopy. The 2021 ATA Guidelines for the Management of Patients with ATC state that total or subtotal resection of the thyroid gland is recommended for patients with limited ATC (stages IVA, IVB) who are expected to achieve an R0 or R1 resection, as well as central and lateral neck lymph node dissection. If there is tracheal or esophageal invasion, local regional resection may be considered for palliative care opportunities (8). Patients with stage IVC ATC do not benefit from surgery for prolonging the patient’s survival, considering that tumor resection may destroy the esophagus and trachea. Therefore, other palliative treatments, including radiotherapy and systemic therapy, are available for these patients.

For this patient, we considered surgical treatment unsuitable. Because of the patient’s advanced age, the risk of surgery was high, and the tumor had extensively invaded the anterior end of the trachea. Combined with the will of the patient and family (refusal of radiotherapy and chemotherapy), we finally chose ultrasound-guided RFA of the thyroid tumor for palliative care. The case underwent ultrasound-guided RFA with tumor shrinkage of 20–30% and relief of neck compression symptoms. Moreover, the results of the follow-up within three months after the procedure did not observe any other uncomfortable symptoms in the patient, suggesting the effectiveness and safety of this treatment option. To the best of our knowledge, this is the first case of palliative care by RFA in a patient with advanced ATC.

RFA is a kind of thermal ablation treatment method, that has been widely used in the treatment of solid tumors of the liver, breast, pancreas and thyroid, etc. (9, 10). The principle of RFA is to apply the energy generated by alternating current (AC) with an oscillating frequency of 200–1200 Hz to make the polar molecules and ions in the tissues vibrate around the electrodes by the change of direction of AC, friction, and then heat is generated, which is then transferred to the tissues through the adjacent electrodes and causes irreversible coagulation, degeneration and necrosis of local tissues and cells. Then, through the heat conduction of neighboring electrodes, the local tissue cells undergo irreversible coagulation, degeneration, and necrosis. After thermal ablation, the coagulated necrotic tissue is gradually absorbed and becomes smaller, and the clinical symptoms caused by the nodules are also significantly improved. Thermal ablation technology is safe, effective, and minimally invasive, especially when the patient refuses surgery, has a high demand for aesthetics, or has contraindications to surgery, RFA serves as an alternative treatment. The advantages of RFA in these aggressive malignancies primarily include its minimally invasive nature, potential for rapid symptomatic relief, and applicability in patients who are ineligible for surgery or radiotherapy. In 2001, Dupuy et al. used RFA for the first time in the treatment of the local recurrence of differentiated thyroid cancer (11). Subsequently, domestic and international studies on the application of RFA in benign thyroid nodules and recurrent thyroid cancer have gradually increased, and corresponding guidelines and consensus have been developed (12–15). Due to the possibility of residual cancerous tissue from incomplete tumor ablation, infiltration of the nodule into surrounding tissues, and occult lymph node metastasis, it is now generally accepted that RFA should be avoided in primary thyroid cancer that is feasible to treat surgically. The complication rate of RFA is about 3.3%, which mainly includes voice alteration, brachial plexus injury, tumor rupture, permanent hypoparathyroidism, thyroid dysfunction, local hemorrhage, skin burns, and thyroid dysfunction. disorders, local hematoma, skin burns, and vomiting (16, 17). In our experience, the ablation area is chosen as the area of enhancement of the thyroid nodule on preoperative ultrasonography, and the area without enhancement needs to be avoided. Of course, the limitations of ablation are the limited coverage of the area by a single ablation and the need for multiple procedures to completely cover the mass when the mass is large. The efficacy of RFA in poorly differentiated/undifferentiated cancers is not well-established in long-term disease control, as these tumors are highly aggressive and tend to infiltrate surrounding structures rapidly. All these were fully informed before the operation, and the patient and his family expressed their understanding. Fortunately, the follow-up of this patient showed a satisfactory outcome with no associated complications.

For patients with stage IVb and IVc ATC that cannot be completely resected by surgery, the current mainstream palliative therapy mainly includes radiotherapy and chemotherapy. External beam radiation therapy can reduce the tumor volume, decrease the risk of local complications and metastasis, enable some patients to gain access to surgery and prolong patient survival (18, 19). Chemotherapy is also an important tool for patients with advanced metastasis of ATC or tumors that cannot be surgically resected before obtaining targeted therapy. The ATA Guidelines for the Management of Patients with ATC from 2021 say that cytotoxic chemotherapy with drugs that contain paclitaxel (paclitaxel or docetaxel) and also with anthracyclines (adriamycin) or platinum (cisplatin or carboplatin) should be used (8). Due to the rapid progression of ATC, radiotherapy or chemotherapy alone is not sufficient. In a study by Prasongsook et al, the lifetime local control rate in patients with surgery combined with chemotherapy and radiotherapy was 90%, and systemic chemotherapy usually precedes radiotherapy after surgery, taking into account factors such as postoperative wound healing (20).

With the development of individualized precision therapy, targeted therapy and immunotherapy have become hot research topics in recent years. It has been found that ATC cells can evade immune surveillance by remodeling the tumor immune microenvironment. Immunotherapeutic strategies represented by targeted blockade of the immune checkpoint PD-1/PD-L1 have been preliminarily demonstrated to be beneficial for ATC patients, especially the combination of molecularly targeted inhibitors and kinase inhibitors with immunotherapy (21–23). For stage IVc ATC patients with mutational deletion of other targets, immune checkpoint inhibitors can be used as their first-line or follow-up therapy if they have high PD-L1 expression (8). Therefore, target drugs for key signaling pathways as well as combination drugs may bring new hope for ATC treatment. Currently, guidelines recommend a multimodal treatment regimen combining surgery with radiotherapy for patients with ATC, such as postoperative chemotherapy with paclitaxel or docetaxel combined with anthracyclines, and external beam radiation therapy (5, 24). However, the overall prognosis of the patient population remains poor due to the highly aggressive nature of ATC.

## Conclusion

For the first time, we provided palliative treatment by RFA for elderly patients with advanced ATC. This case exemplifies a new application and success of this classical approach. It suggests that RFA has a good therapeutic effect, and we hope that it will provide new ideas for the treatment of ATC in the future.

## Data Availability

The original contributions presented in the study are included in the article/supplementary material. Further inquiries can be directed to the corresponding author.
